# Gas Sensor Properties of (CuO/WO_3_)-CuWO_4_ Heterostructured Nanocomposite Materials

**DOI:** 10.3390/ma18122896

**Published:** 2025-06-18

**Authors:** Michael Castaneda Mendoza, Carlos A. Parra Vargas, Miryam Rincón Joya, Adenilson J. Chiquito, Angela M. Raba-Páez

**Affiliations:** 1 Grupo Física de Materiales—GFM, Universidad Pedagógica y Tecnológica de Colombia, Avenida Central Del Norte 39-115, Tunja 150003, Boyacá, Colombia; michael.castaneda@uptc.edu.co (M.C.M.); carlos.parra@uptc.edu.co (C.A.P.V.); 2 Grupo de Física Mesoscópica, Universidad Nacional de Colombia, Carrera 30 Calle 45-03, Bogotá 111321, Cundinamarca, Colombia; 3 NanoLab, Departamento de Física, Universidade Federal de São Carlos, Rodovia Washington Luiz km 235, São Carlos 13565-905, São Paulo, Brazil; chiquito@df.ufscar.br; 4 Grupo de Investigación en Materiales Poliméricos—GIMAPOL, Universidad Francisco de Paula Santander, Avenida Gran Colombia 12E-96, Cúcuta 540003, Norte de Santander, Colombia; angelamercedesrp@ufps.edu.co

**Keywords:** gas sensor, heterostructure, WO_3_, CuO, CuWO_4_, acetone, methanol

## Abstract

In this work, we report the evaluation of a (CuO/WO_3_)-CuWO_4_ heterostructured system as a methanol and acetone gas sensor in different configurations, contrasted with the pure oxides CuO and WO_3_. The samples were synthesized using a modified precipitation route followed by a single thermal treatment step to induce multiphase simultaneous crystallization. The structural characterization by XRD showed that all the materials presented the formation of monoclinic CuO and WO_3_ and triclinic CuWO_4_. No additional phases were detected in the samples, and a reduction in the crystallite size of the CuO phase after the crystallization in the heterostructured system was observed. FE-SEM analysis made it possible to directly observe the morphology and the structures of the samples at the nanometer scale, showing a heterogeneous grain formation and supporting the formation of a heterostructure. UV-Vis DRS was used to study the optical properties of the materials, and the presence of two optical band gaps was successfully determined, which provides further evidence of heterostructure formation via this modified synthesis route. The variation in the resistance of the materials was observed in the presence of methanol and acetone vapors, where the heterostructure exhibited a substantial change in performance in the configuration with 40% copper precursor (Cu40:W60), the sample that presented the highest response as a sensor against these VOCs. To our knowledge, this is the first time that this system has been reported as a gas sensor, using the multiple configurations of the (CuO/WO_3_)-CuWO_4_ heterostructured system.

## 1. Introduction

Currently, there is a significant scientific interest in the quantifying pollutant compounds present in different essential natural resources such as water and air. The accelerated development of human activity and industrial development has led to an increase in the emission of highly toxic pollutants to humans. Although these compounds are important in the early stages of industrial processes, when they are released or escape from the processes, they result in potentially harmful and toxic compounds for any living organism. Examples of these gases include ethanol, methanol, acetone, and benzene, among others, generally classified as volatile organic compounds (VOCs). Exposure to concentrations above permissible limits can lead to health problems and even death [1].

Tungsten oxide (WO_3_) is among the most cost-effective n-type semiconductors, showing promising performance in many applications, such as solar cells, sensors, photoelectrochemical water splitting, photocatalysis, and superconductivity, among others [2,3,4]. Due to its narrow indirect band gap, between 2.4 and 3.0 eV [5,6], it can absorb visible light more efficiently compared to other commonly studied photocatalytic semiconductors [7,8,9]. However, the electron–hole recombination rate tends to be higher compared to other semiconductors. On the other hand, copper oxide (CuO) is one of the most widely studied p-type oxides. It has an indirect band gap between 1.2 and 2.1 eV [10]. In order to overcome some of the transport disadvantages of many semiconductors and increase their photocatalytic efficiency, heterostructured oxide systems have been developed to reduce charge carrier recombination. Specifically, CuO has been used as a counterpart (p-type) for WO_3_ (n-type) in various applications [11,12,13,14,15,16], such as a catalyst in dye degradation processes, organic gas sensors, and solid oxide batteries [14,17,18]. CuO-WO_3_ nanofibers (NFs) fabricated via electrospinning method and tested for sarin gas sensor detection demonstrated that the detection performance of WO_3_-CuO NFs is better compared to WO_3_ and pure CuO and showed excellent selectivity toward trace amounts of sarin gas at room temperature from among several volatile organic compounds [12]. A hydrothermal synthesis of CuO-WO_3_ nanocubes for H_2_S gas sensing showed substantially greater and faster sensitivity and recovery than those of individual pure samples [19]. The increased surface area of the flower-like morphology, compared to the basic WO_3_–CuO composites, led to a higher density of active sites, which are essential for sensor performance. This morphological modification significantly enhanced H_2_S detection [16].

Copper tungstate (CuWO_4_) could be a key component in enhancing the efficiency of heterojunctions, particularly in CuO/WO_3_ systems. It promotes efficient electron transport and reduces recombination at the interface, while minimizing interfacial defects and enhancing charge carrier separation. CuWO_4_ can form high-quality interfaces when synthesized via in situ methods, reducing recombination and boosting charge transfer. Studies have shown that CuWO_4_-based systems exhibit superior performance in photoelectrochemical cells and gas sensors. CuWO_4_ also enhances light absorption and increases the active catalytic surface area, making it a valuable addition to multicomponent systems. Further exploration could lead to significant improvements in material performance [20]. The (CuO/WO_3_)-CuWO_4_ system has not been previously reported or systematically investigated for gas sensing applications.

In this work, pristine CuO, WO_3_, and (CuO/WO_3_)-CuWO_4_ were synthesized via a modified precipitation step. The materials were characterized using X-ray diffraction (XRD), Fourier transform infrared (FTIR) spectroscopy, scanning electron microscopy (SEM), and UV-Vis diffuse reflectance spectroscopy (UV-Vis DRS). XRD analysis, with the Rietveld refinement step, allowed the phase identification, quantification, and determination of lattice parameters. FTIR spectra provided confirmation of vibrational modes associated with Cu–O and W–O bonds. FE-SEM was used to directly observe the morphology at the nanometer scale, and DRS enabled the estimation of optical band gaps using the Kubelka–Munk model. Finally, the samples were tested as gas sensors by exposing the materials to methanol and acetone vapors in a controlled nitrogen gas flow. This study introduces, for the first time, the evaluation of various configurations of the (CuO/WO_3_)-CuWO_4_ heterostructure as a gas sensor.

## 2. Materials and Methods

### 2.1. Synthesis

**Figure 1:**
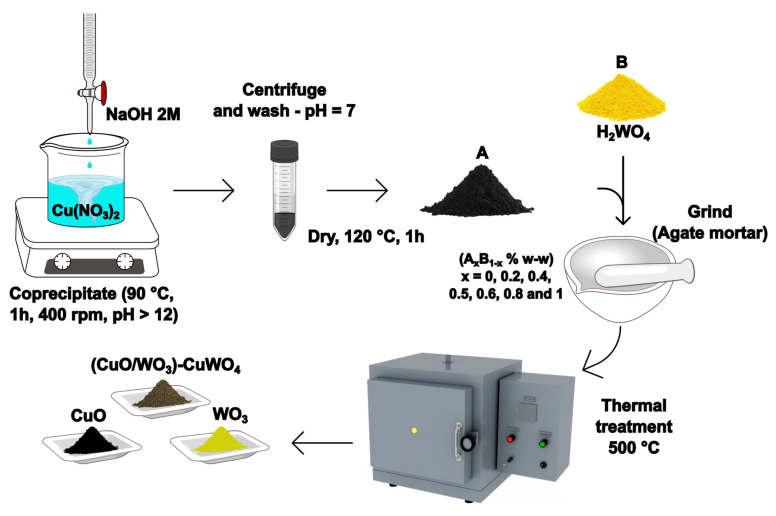
Schematic general synthesis process, where A = copper precursor, B = tungsten precursor, and x corresponds to the mass fraction of the Cu−based precursor (A) and W−based precursor (B). For example, A_0.5_B_0.5_ represents a 50:50 weight ratio.

The general synthesis process is illustrated in Figure 1. Initially, a stoichiometric quantity of high-purity copper (II) nitrate 3-hydrate (Cu(NO_3_)_2_⋅3H_2_O, 98–103%, PanReac AppliChem ITW reagents, Germany) was dissolved in double-distilled water (DDW). Subsequently, the pH was adjusted to 12 using a 2M sodium hydroxide (NaOH, 98%, PanReac AppliChem ITW reagents) solution, added dropwise. The resulting solution was aged for 0.5 h at 90 °C and 400 rpm. The resulting precipitate was centrifuged and washed with DDW until it reached a pH of 7. The resultant black material was dried for 1 h at 120 °C (A in Figure 1), then was ground into a black powder using an Agate mortar.

For the CuO sample, a portion of the material was heated in a Terrigeno D8-2471 oven (Colombia), with the temperature gradually increasing at a rate of 3 °C/min until reaching 500 °C, and maintained for 2 h. In the case of the heterostructure samples, a portion of the initial black powder was combined with a stoichiometric amount of tungstic acid (B Figure 1) (H_2_WO_4_, 99%, Sigma Aldrich, USA) to obtain mixtures containing 80, 60, 50, 40, 20, and 0 w/w % of copper precursor. The resulting samples were subjected to the same calcination ramp: 500 °C for 2 h, based on thermal stability and optimized as reported previously [21]. In the case of the WO_3_ sample, the same procedure was followed, omitting the copper precursor addition step.

Following the procedure outlined in Figure 1, a set of 7 different samples were obtained, which were labeled as follows in Table 1.

### 2.2. Characterization

The structural characterization of the samples was conducted through XRD analysis to identify the crystal structure and quantify the phase composition. An X’pert Pro Panalytical diffractometer (Amsterdam, The Netherlands) with a real-time multiple strip (RTMS) detector in Bragg–Brentano mode (Cu Kα radiation of λ = 1.542 Å) was used; the 2θ angle range was set between 20 and 80° with a step size of 0.026°. FTIR spectroscopy was used to confirm the presence of characteristic vibrational bands on the surface of the samples. A Shimadzu Prestige-21 IR spectrometer (Kyoto, Japan) with a resolution of 8 cm^−1^ and 8 runs was used for these measurements. Morphological details were observed through FE-SEM using a Tecnai F20 Super-Twin TMP microscope (United States) operating at an accelerating voltage of 200 kV, and the samples were dispersed in absolute ethanol and deposited on copper grids with 200 Mesh Lacey/Carbon membranes. Particle size distribution was estimated from FE-SEM images using ImageJ (1.53j) software by measuring at least 150 particles per sample. Finally, the determination of the band-gap energy of the samples was performed from the reflectance spectra obtained by UV-Vis DRS with a UV-Vis Cary 5000 spectrophotometer (Malaysia) in diffuse reflectance mode, using the Kubelka–Munk based on the absorbance data obtained.

### 2.3. Methanol and Acetone Sensing Test

The sensor responses were evaluated by depositing a mixture of the powders dispersed in isopropanol on substrates with interdigitated electrodes. The sensors were introduced in a stainless-steel test chamber for the sensing tests. The experimental bench for the electrical characterization of the sensors allows measurements to be carried out in a controlled atmosphere. Gases from certified cylinders were diluted in nitrogen (60 sccm) to the desired concentrations using mass flow controllers. Sensing measurements were carried out in the temperature range of 150–400 °C, with steps of 50 °C, under a gas (methanol or acetone) total stream of 30 sccm, collecting sensor resistance data in two-point mode under the applied bias of +10 V. A digital multimeter was used for data acquisition. The gas response is defined as the ratio R_a_/R_g_, where R_a_ is the electrical resistance of the sensor in dry air and R_g_ is the resistance at different methanol and acetone concentrations [22].

## 3. Results and Discussion

### 3.1. Structural Characterization

**Figure 2:**
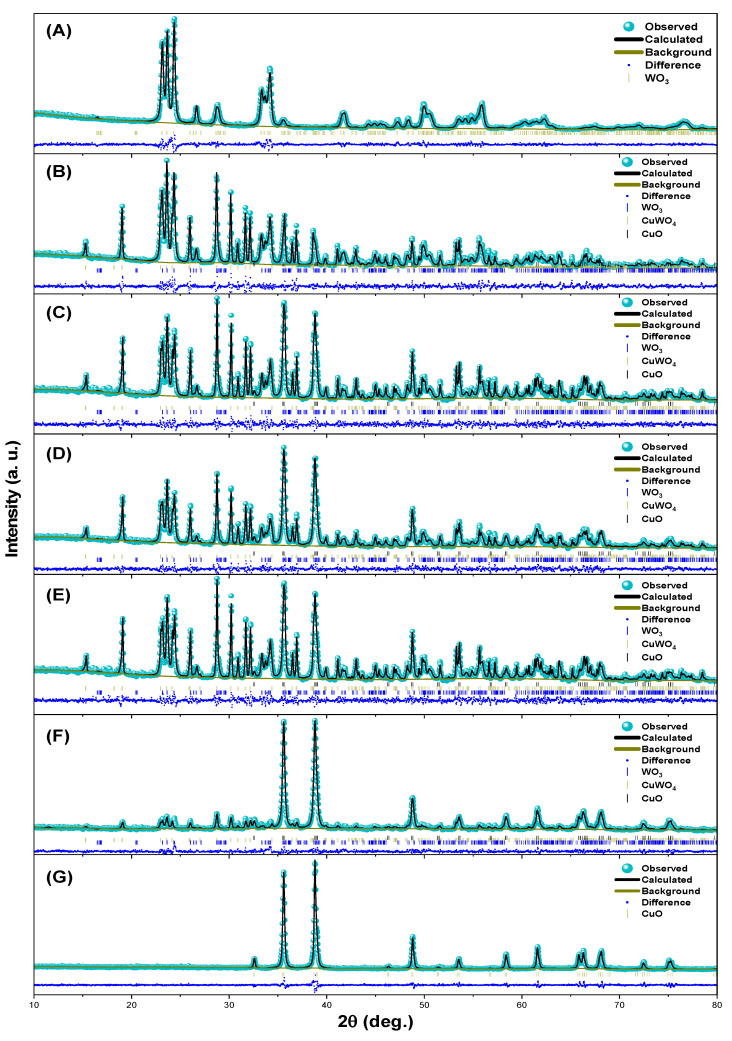
The Rietveld refinement plots of the WO_3_ (**A**), Cu20:W80 (**B**), Cu40:W60 (**C**), Cu50:W50 (**D**), Cu60:W40 (**E**), Cu80:W20 (**F**), and CuO (**G**) samples.

The structural properties, crystalline structure, and the phase composition of the samples were analyzed through XRD analysis. Rietveld refinement was conducted using GSAS-II software [23,24]. Figure 2 shows the plots generated from the refinement of the samples. The Rietveld adjustment showed a good agreement between the theoretical model and the experimental data, as confirmed by the low chi-squared ($\chi^{2}$) value (Table 2). This confirms the quality of the adjustment and the accuracy of phase identification. The CuO and WO_3_ contrast samples did not show the presence of secondary phases. All the samples with a copper precursor showed characteristic peaks around 35.5° and 38.8°, attributed to the diffraction planes, (−111), (111), respectively, and indexed to the monoclinic crystalline phase of CuO with PDF No. 01-072-0629 and the *C*2/*c* (15) space group. The presence of these characteristic peaks was reported previously [25,26,27].

Additionally, in the samples with a tungsten precursor, characteristic signals were observed around 23.1°, 23.6°, and 24.4° corresponding to the diffraction planes, (002), (020), and (200), respectively, and these signals are consistent with the monoclinic WO_3_ phase, PDF No. 01-072-1465 with the *P*21/*n* (14) space group system. These results are comparable to those reported by Kang et al. [28]. Finally, in all the heterostructure samples, the CuWO_4_ triclinic phase was observed, and characteristic signals around 19.2, 28.3, and 30.5° indexed to the diffraction planes, (100), (−1−11), and (111), respectively, confirm the presence of this phase, which matches PDF No. 01-070-1732 and the *P*-1 (2) space group, previously reported at this crystallization temperature as α-CuWO_4_ [29]. This additional phase is commonly formed due to the coexistence of copper and tungsten precursors. Its formation results from a secondary reaction between these compounds, occurring concurrently with the formation of CuO and WO_3_ phases [30]. The presence of these distinctive peaks confirms the coexistence of all the phases in the synthesized samples. Overall, the existence of distinct phases in the samples indicates the formation of a multicomponent system. This coexistence could indicate an interaction between the phases, potentially influencing the material properties [15,20].

**Figure 3:**
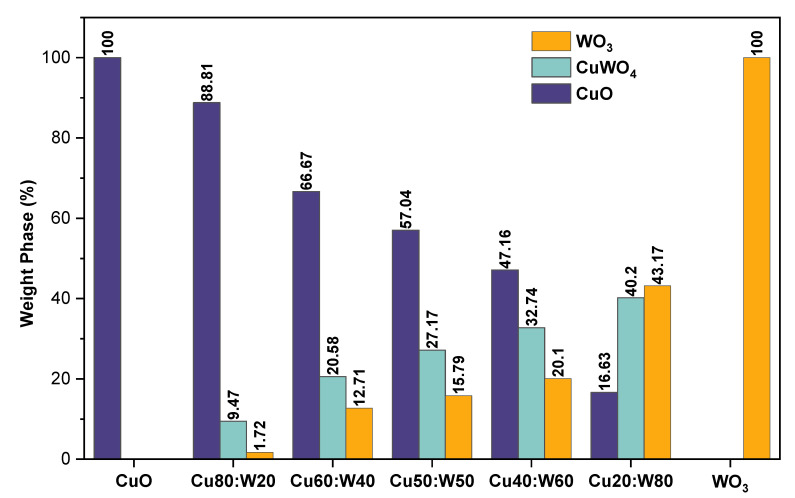
Weight percentage of the phases in the synthesized samples.

The weight percentage of the phases in the synthesized samples, presented in Figure 3, reveals a clear variation in the phase composition among the samples. In Cu80:W20, monoclinic CuO was the majority phase with 88.81%, then a small proportion of CuWO_4_ (9.47%) and trace amounts of WO_3_ (1.72%). A significant rise in the percentage of the WO_3_ phase is seen when the copper precursor concentration decreases, reaching 43.17% in the Cu20:W80 and 100% in the pure WO_3_ sample. Conversely, the CuWO_4_ phase rises gradually to reach 40.2% in the Cu20:W80 sample, suggesting that the CuWO_4_ content stabilizes in the samples with lower CuO content. These differences in phase composition can directly influence the physicochemical properties of materials, particularly their sensitivity as gas sensors, given that the WO_3_ phase is known for its good response in this type of application [31,32]. The observed phase evolution suggests that controlling the initial composition is a key factor in tuning the final properties of the materials. In addition, the obtained lattice parameters (Table 2) suggest slight variations in the crystal structure of the phases, which could be related to the generation of structural defects or structural distortions inherent to the synthesis methods used.

Additionally, the average crystallite size (*L*) was determined using the Debye–Scherrer equation. In Equation (1), the Scherrer constant K=0.94, λ=0.1540598 nm, β is the full width at half maximum (FWHM), and θ is the Bragg angle. In all the samples, the maximum intensity peak of each phase in the diffractogram was selected for crystallite size estimation. In addition, Rietveld L was also estimated in both perpendicular (L⊥) and parallel (L∥) directions with Equations (2) and (3), based on the Lorentzian component parameters (LX and *ptec*) from GSAS refinement. *K* and *λ* correspond to Scherrer parameters [33,34].

(1) $$L=\frac{K\lambda }{\beta cos\theta }$$

(2) $$L⊥=\frac{1800\;K\lambda }{\pi LX}$$

(3) $$L∥=\frac{1800\;K\lambda }{\pi (LX+ptec)}$$

**Figure 4:**
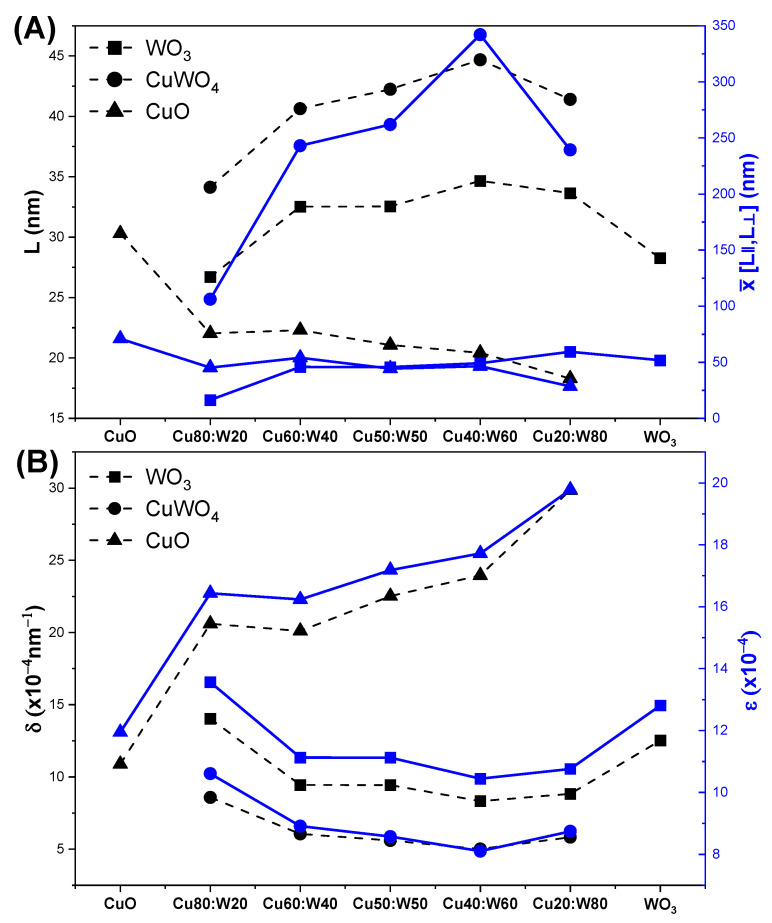
Variation in Scherrer and Rietveld crystallite size (**A**), dislocation density, and microstrain (**B**) in synthesized samples.

Figure 4A shows the variation in Scherrer and Rietveld L for CuO, WO_3_, and CuWO_4_ phases between the synthesized samples. The parallel and perpendicular Rietveld L mean values show similar trends to the results obtained by Scherrer. The results show that CuO phase crystallite size decreases with lower copper precursor content, with minimum values observed in Cu20:W80. WO_3_ phase crystallite size remains relatively constant between Cu80:W20 and Cu20:W80, with the lowest values found in Cu80:W20 and pure WO_3_ samples.

For the CuWO_4_ phase, crystallite size increases until reaching a maximum in the Cu40:W60 sample, then decreases in samples with lower copper content, such as Cu20:W80. The Rietveld refinement values provide additional information on particle anisotropy, with a noticeable increase at intermediate compositions, especially at Cu40:W60, where both CuWO_4_ and WO_3_ reach maximum crystallite sizes. This suggests that crystallite growth is more pronounced in this specific composition, possibly due to phase stabilization or interactions among the constituent oxides. The analysis of crystallite sizes demonstrates the influence of material composition on structural properties, particularly for the WO_3_ phase, known for its good gas detection performance [35].

The dislocation density (δ) and microstrain (ε) shown in Figure 4B were also determined with Equations (4) and (5), respectively, as follows in [36]. ε represents lattice resulting from imperfections or irregularities in the atomic structure [37]. The CuO and WO_3_ phases showed the highest microstrain, possibly due to their nanocrystalline nature and intrinsic properties. This strain could be favorable for applications like gas sensing and photocatalysis, where surface defects and lattice distortions enhance the reactivity and sensitivity [35]. δ is inversely related to crystallite size, affecting both mechanical and electronic properties. CuO shows a higher dislocation density compared to CuWO_4_ and WO_3_, indicating a higher density of internal defects associated with its smaller crystallite size. These dislocations can enhance mechanical strength and act as charge carrier traps, affecting the electrical properties of CuO and metal oxides in general, especially in semiconductor devices [38,39].

(4) $$\delta =\frac{1}{L^{2}}$$

(5) $$\varepsilon =\frac{B\cos\theta }{4}$$

**Figure 5:**
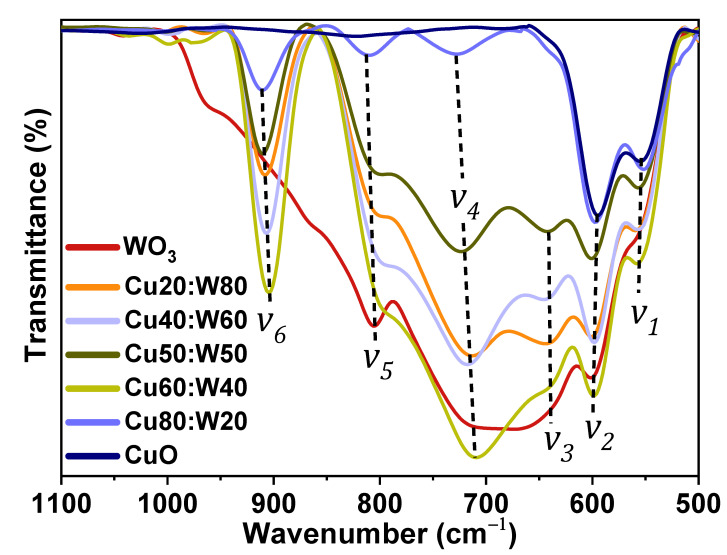
FTIR spectra of the synthesized samples in the region of 500 to 1100 cm^−1^.

The vibrational modes $\left(v\right)$ of the synthesized materials were determined through FTIR spectroscopy. The experimental spectra, presented in Figure 5, reveal intense peaks in the region of 500–1100 cm^−1^. The red shifts (lower wavenumber displacements) and blue shifts (higher wavenumber displacements) presenting the different $v_{n}$, are attributed to dislocations and structural defects in the materials [40]. This variation is consistent with the structural distortions observed in the XRD analysis. These results provide insight into the bonding environment within the heterostructures. The presence of multiple metal–oxygen stretching modes indicates the coexistence of Cu-O and W-O bonds; the observed vibrational bands are reported and assigned in Table 3. The $v_{6}$ band, corresponding to W-O stretching in tetrahedral coordination, confirms the successful formation of the ternary structure. This analysis supports the phase identification obtained from the XRD results.

### 3.2. Morphological Characterization

**Figure 6:**
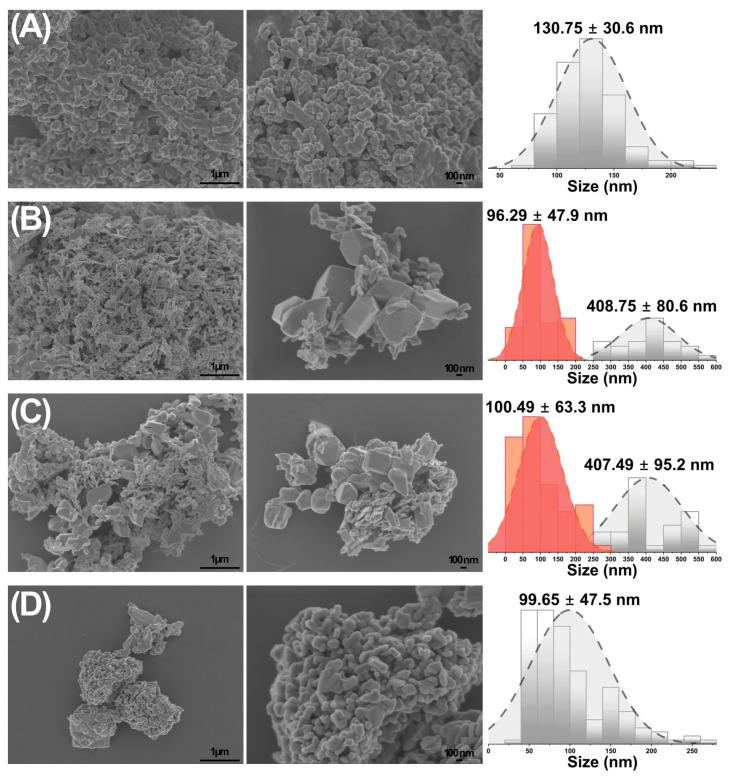
FE-SEM images at 20–50 kx and particle size distributions estimated from SEM image analysis using ImageJ of (**A**) CuO, (**B**) Cu60:W40 (Orange—Small Particle Size; Gray—Large Particle Size (nm)), (**C**) Cu50:W50, and (**D**) WO_3_ samples.

FE-SEM images (Figure 6) show the morphology of the CuO, WO_3_, Cu60:W40, and Cu50:W50 samples. The morphology of the samples varies significantly, and two predominant particle size distributions were observed for the copper- and tungsten oxide-based samples except for Cu80:W20. The CuO sample (Figure 6A) shows a porous, network-like structure composed of densely agglomerated nanoparticles. Sample Cu60:W40 (Figure 6B) shows more compact and well-defined polyhedral grains, attributed to the presence of the CuWO_4_ phase, which appears exclusively in samples containing the CuWO_4_ phase; agglomerated particles were also observed in the sample. The Cu50:W50 sample (Figure 6B) exhibits granular structures similar to those of Cu60:W40., and this morphology was constant across all the heterostructures samples, except Cu80:W20. These findings confirm that the phase composition directly influences the morphology of the phases as the composition of the system changes, and they also indicate that co-crystallization affects structural features. The sample WO_3_ (Figure 6D) shows irregular nanoparticle agglomeration with smooth surfaces. These morphological changes reflect differences in phase distribution, synthesis conditions, and particle growth mechanisms between samples, which influence both texture and particle size distribution. The particle size distributions for the samples are reported in Table 4.

The small particle sizes range from 80.9 nm to 130.8 nm, with the CuO sample exhibiting the largest average size (130.8 ± 30.6 nm) and the Cu80:W20 sample showing the smallest average (80.9 ± 28.3 nm). Large particles, on the other hand, exhibit more uniform sizes throughout the samples, ranging in size from 407.1 nm to 426.5 nm. For example, samples Cu60:W40, Cu50:W50, and Cu40:W60 contain large particles with comparable diameters around 407.1 nm, suggesting that particle growth in these compositions may be stabilized. This bimodal particle size distribution behavior might be attributed to the synthesis conditions and the heterogeneity of the samples, with various components and phases leading to the formation of particle sizes of varying sizes. Larger particles may result from the agglomeration or coalescence of smaller crystallites during growth, whereas the smaller particles correspond to isolated crystalline domains [46].

### 3.3. Optical Properties

**Figure 7:**
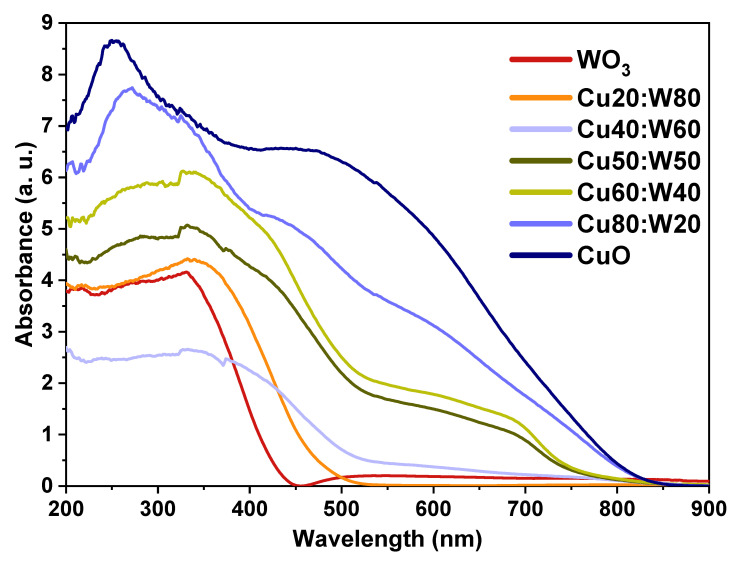
The UV-Vis DRS spectra of the synthesized materials.

The optical properties of the obtained samples were analyzed via UV-Vis DRS. Figure 7 presents the absorbance behavior of the samples, where the CuO sample exhibits the broadest absorption range, which extends approximately from 200 to 800 nm with a strong peak at 300 nm, and undergoes a red shift as the CuO content decreases in the heterostructured samples. In these, the absorption range broadens with increasing CuO content, with the Cu80:W20 and CuO samples showing the widest absorption range. This behavior suggests that CuO has a greater ability to absorb light along the visible spectrum due to its narrower band gap compared to the WO_3_ and CuWO_4_ phases. The WO_3_ sample exhibited a limited absorption range, which could be attributed to the absence of oxygen vacancies or defect states [47], which is consistent with previous reports [48]. Previous studies have shown that the formation of the CuWO_4_ phase also induces a red shift in the optical absorption spectrum. This shift is attributed to the narrowing of the band gap, which enhances light absorption at longer wavelengths, particularly in the visible region [20,49]. In general terms, these findings demonstrate that the heterostructure generated by different oxides outperforms the individual oxides in terms of optoelectronic application due to the optimized optical response within the visible spectrum.

**Figure 8:**
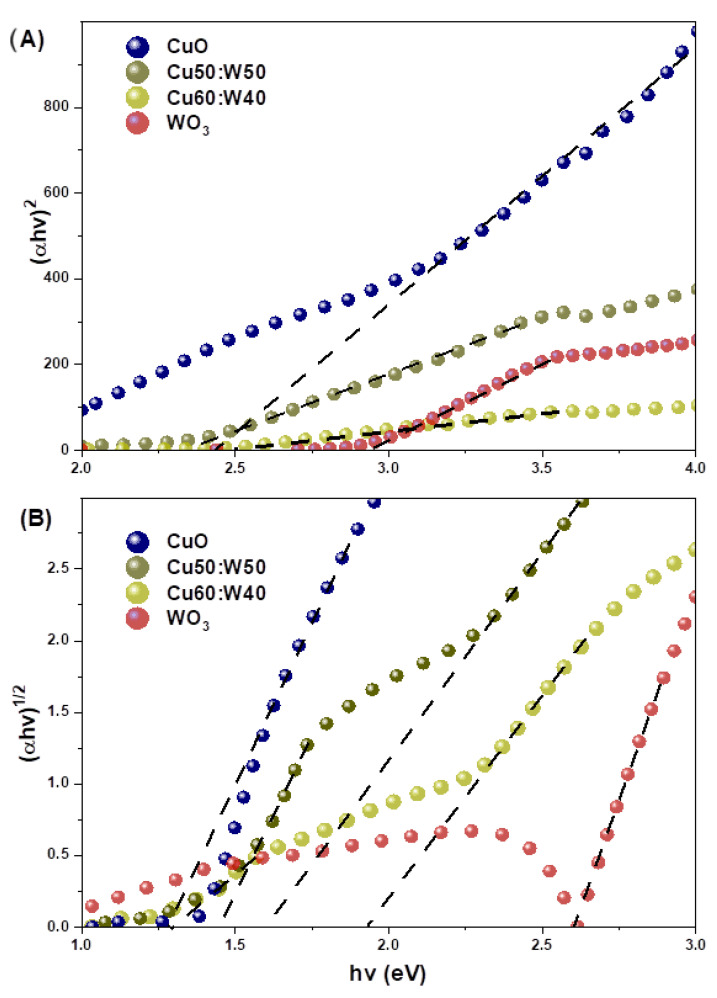
(**A**) Direct (αhv)^2^ and (**B**) indirect (αhv)^1/2^ transitions vs. photon energy. The dotted line indicates the linear fit used to estimate the optical band gap.

The optical band gap energy ($E_{g}$) of the samples was determined via UV-Vis DRS, using the Kubelka–Munk transform method, which assumes absorption and scattering as first-order phenomena [50,51]. The line segment x-intercept of the ${\left(\alpha hv\right)}^{1/n}$ versus the energy of the incoming photon (hv) (Figure 8), derived from Equation (6), was used. In this, C is a proportionality constant, and n is a variable coefficient that changes based on the kind of electronic transition; direct permitted transitions and indirect permitted transitions have n = 1/2 and n = 2, respectively. h is the Planck constant and ν is the light frequency of the incident light [52,53,54].

(6) $$\alpha hv=C{\left(hv-E_{g}\right)}^{n}$$

The approximate direct and indirect band-gap values of the samples are displayed in Table 5. The identification of different indirect band gaps suggests the coexistence of multiple semiconductor phases. This result, together with structural and morphological evidence, supports the formation of a heterostructure. From the CuO to Cu40:W60 samples, two different band gaps were observed, although three semiconductor phases coexist in the heterostructured samples (CuO, WO_3_, and CuWO_4_), and only two distinct optical transitions were observed in the UV–Vis DRS analysis. This is attributed to the close proximity of the indirect band gaps of WO_3_ (2.44 eV) and CuWO_4_ (~2.35 eV [20,55]), which likely leads to overlapping absorption features that cannot be individually resolved in Kubelka–Munk plots [56,57]. As a result, a single indirect transition is observed in that region. In contrast, the CuO phase exhibits a lower band gap (1.28 eV), which is clearly distinguishable. Therefore, the two observed band gaps correspond to CuO ($E_{g1})$ and the combined contribution of WO_3_/CuWO_4_ ${(E}_{g2})$. The band gaps of some heterostructures were lower than those of the individual oxides after the simultaneous crystallization step, suggesting a potential extension of the system’s light absorption range. This suggests that the heterostructured samples are capable of absorbing visible light [15]. The optical response in the heterostructure is often enhanced by a reduced optical band gap. This narrower band gap makes it easier for electron–hole pairs to form and move between CuO, CuWO_4_, and WO_3_, which may improve the synergistic optical behavior of the combined oxides [56]. Accordingly, the CuO and Cu40:W60 samples exhibit promising optical performance.

### 3.4. Gas Sensing Test

**Figure 9:**
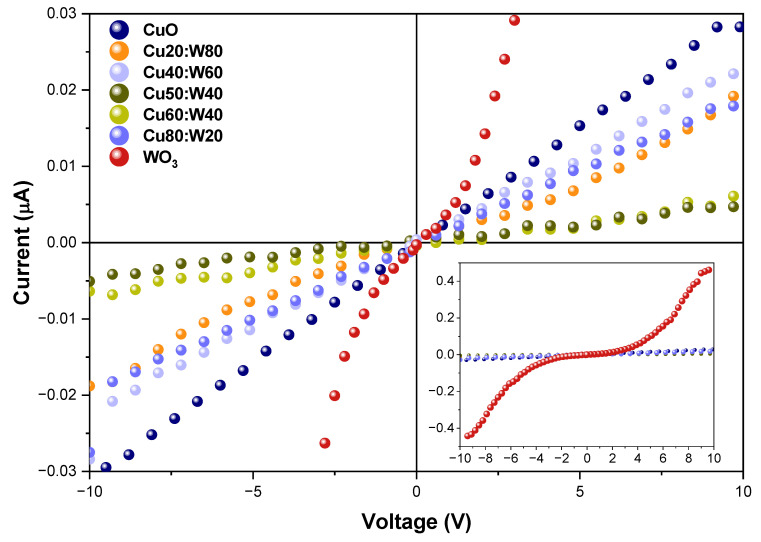
Current in function of voltage curves of synthesized samples at 300 K.

The I–V (current vs. voltage) curves presented in Figure 9 exhibit typical ohmic behavior in the CuO, Cu20:W80, Cu40:W60, Cu50:W50, Cu60:W40, and Cu80:W20 samples, indicating low contact resistance and charge transport within the applied voltage range [12,58,59]. A device with this behavior will ensure functionality at low power and with minimum energy consumption. In contrast, the nonlinear behavior of the WO_3_ sample at higher voltages is mainly due to a space charge-limited current mechanism. In this case, for low values of voltage, the current exhibits near-ohmic behavior; however, upon increasing the voltage, charge traps within the material limit current flow, resulting in this phenomenon [60,61].

**Figure 10:**
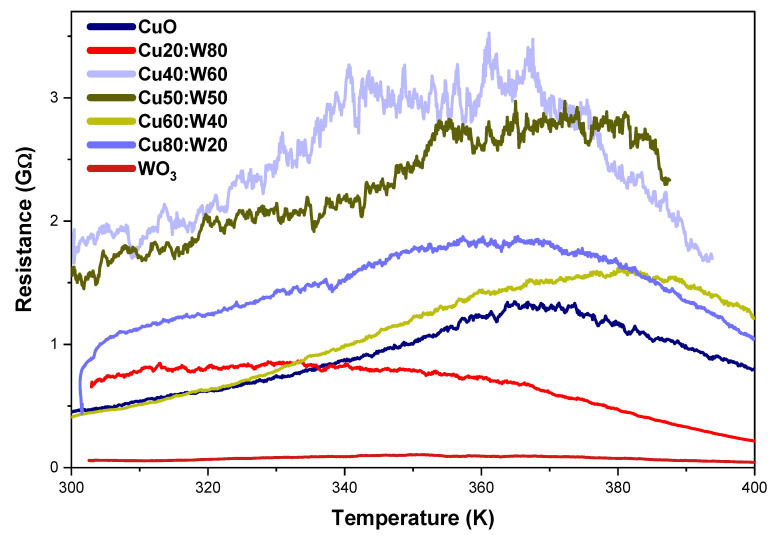
Variation in sample resistance with temperature.

On the other hand, the analysis of resistance as a function of temperature (Figure 10) clearly shows that the resistance decreases with increasing temperature due to the granular nature of the material, which creates multiple potential barriers between grains, as well as depletion layers within the heterostructure channels. The optimum operating temperature of a sensor directly affects its resistivity by balancing the adsorption and desorption of gas molecules on the sensor surface, with higher resistance at moderate temperatures due to oxygen adsorption, whereas at higher temperatures, desorption reduces the sensor response. These findings agree well with metal oxide-based materials, which constitute one of the most studied families of materials in gas sensors due to their high sensitivity and thermal stability [59,62,63].

**Figure 11:**
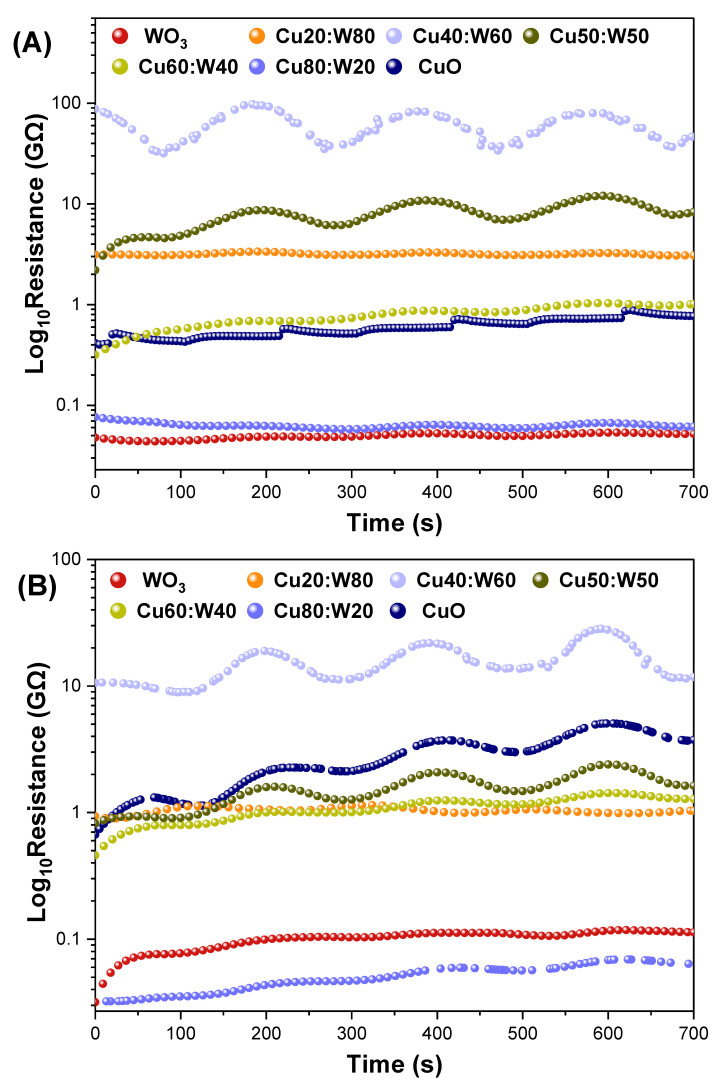
Resistance changes in sample sensors for methanol (**A**) and acetone (**B**) vapors at 350 K.

In the gas sensor analysis, the sample sensors showed a good response to methanol/acetone vapors. Figure 11 shows the resistance profile of all samples exposed to a fixed vapor concentration in N_2_ over time. Exposure to methanol (Figure 11A) and acetone (Figure 11B) induces a decrease in resistance, characteristic of the semiconductor’s response to reducing gases. The novel heterostructure system increases the number active sites, which are essential for gas sensing performance, observing a higher response or resistance change in the Cu40:W60 and Cu50:W50 samples. This enhanced behavior is attributed to the multicomponent nature of the sensor, made from metal oxides, which works on the principle of heterostructures between p-type and n-type materials, such as CuO and WO_3_-CuWO_4_, respectively. In this type of sensor, upon being brought into contact with a p-type material with an n-type material, an electron depletion layer forms in the n-type material, while a hole depletion layer forms in the p-type material, resulting in the formation of a potential barrier at the interface [62].

**Figure 12:**
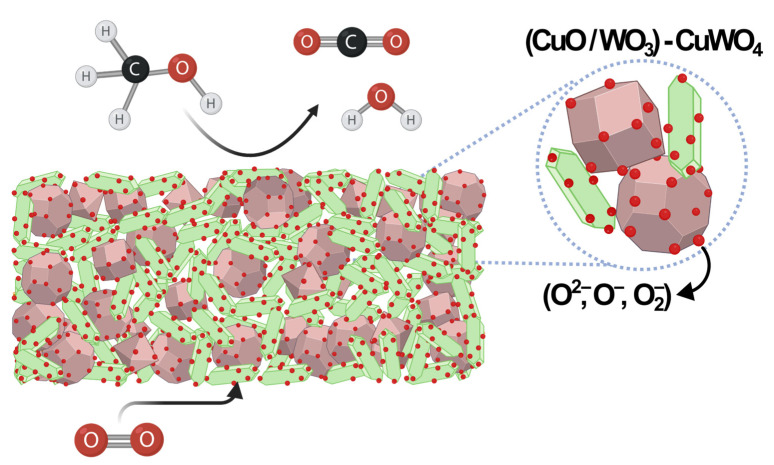
Schematic representation of methanol sensing mechanism in (CuO/WO_3_) − CuWO_4_ heterostructure.

In contact with oxygen, the sensor surface promotes its adsorption, forming adsorbed oxygen species ($O^{2-},O^{-},O_{2}^{-}$), which subsequently capture electrons from the n-type materials (WO_3_ and CuWO_4_), leading to an expansion of the depletion layer and an increase in sensor resistance. When exposed to reducing gases such as acetone and methanol, the oxygen ions combine with such gases, releasing electrons, which reduces both the potential barrier and the depletion layer width, thereby decreasing the resistance. The gases react with the oxygen species on the material surface, producing byproducts such as CO_2_ and H_2_O, among others, depending on the gas’s nature [47,63,64,65]. A schematic representation of this mechanism is shown in Figure 12.

**Figure 13:**
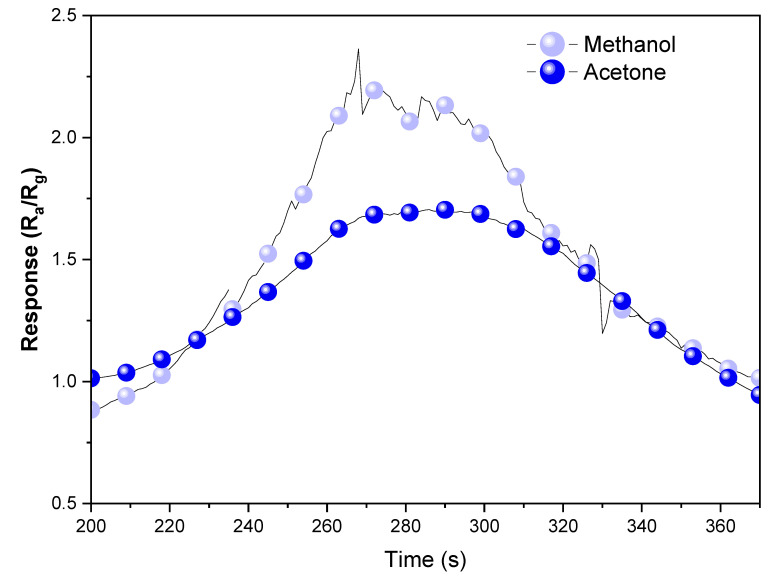
Cu40:W60 sample response in methanol and acetone gas sensor test.

Figure 13 shows how a gas sensor measures the change in resistance from its initial state (Ra) to its state after gas exposure (Rg), enabling the calculation of the sensor response as Ra/Rg for the detected gas. The improved behavior observed in the p–n heterostructure exhibits a faster and more sensitive response and recovery when compared to materials without this type of configuration. For the most responsive material configuration (Cu40:W60), the response to methanol was higher than acetone, 2.2 and 1.7, respectively. This difference is usually reported for semiconductor-based materials [66]. For the CuO nanowires, the ethanol response was reported as 1.5 [67] and 1.4 for methanol [66], and for the WO_3_ thin films, the response was 1.5 for ethanol [68]. The enhanced response of the heterostructured system over the pristine WO_3_ sample and high WO_3_ phase percentage (Cu80:W20) sample shows a superior performance in samples with higher CuO content (p-type component), as previously observed in CuO/WO_3_ heterostructures [69].

## 4. Conclusions

Gas sensing measurements performed on (CuO/WO_3_)-CuWO_4_ heterostructures show promising results in detecting methanol and acetone vapors. The ohmic behavior observed in most samples through the I-V curves indicates low contact resistance and good electrical conductivity, which is essential for the operation of low-power sensors. The heterostructure samples, especially the Cu40:W60 and Cu50:W50 samples, exhibited an improved gas sensitivity, which was tuned by n-type and p-type sensing mechanisms that promote electron transfer between the p-type CuO and n-type WO_3_-CuWO_4_ components. This configuration increases the number of active sensing sites, leading to significant changes in resistance upon exposure to reducing gases such as methanol and acetone. The Cu40:W60 sample exhibited excellent sensitivity, especially toward methanol, with a response ratio of 2.2 compared to 1.7 for acetone. These results highlight the potential application of (CuO/WO_3_)-CuWO_4_ heterostructures in gas sensing due to their high sensitivity, good stability, and rapid response times.

## Figures and Tables

**Table 1:** Sample labeling.

Label	Weight Percentage (w-w %)	
	Copper Precursor (A)	Tungsten Precursor (B)
WO_3_	0	100
Cu20:W80	20	80
Cu40:W60	40	60
Cu50:W50	50	50
Cu60:W40	60	40
Cu80:W20	80	20
CuO	100	0

**Table 2:** Rietveld refinement parameters of the synthesized samples.

	Phase	CuO	Cu80:W20	Cu60:W40	Cu50:W50	Cu40:W60	Cu20:W80	WO_3_
a (Å)	CuO	4.69	4.69	4.68	4.69	4.69	4.68	-
	WO_3_	-	7.50	7.30	7.31	7.31	7.33	7.32
	CuWO_4_	-	4.70	4.70	4.71	4.71	4.70	-
b (Å)	CuO	3.43	3.43	3.43	3.42	3.42	3.40	-
	WO_3_	-	7.60	7.52	7.53	7.53	7.56	7.54
	CuWO_4_	-	5.84	5.83	5.84	5.84	5.83	-
c (Å)	CuO	5.13	5.14	5.13	5.14	5.13	5.12	-
	WO_3_	-	7.65	7.67	7.69	7.69	7.72	7.71
	CuWO_4_	-	4.88	4.87	4.88	4.88	4.88	-
V (Å^3^)	CuO	82.52	82.69	82.35	82.44	82.28	81.47	-
	WO_3_	-	436.05	421.05	423.29	423.29	427.80	425.54
	CuWO_4_	-	133.95	133.44	134.23	134.23	133.72	-
α (°)	CuO	90						-
	WO_3_	-	90					
	CuWO_4_	-	91.67	91.66	91.66	91.65	91.66	-
β (°)	CuO	99.52	99.52	99.55	99.54	99.54	99.60	-
	WO_3_	-	90.42	90.50	90.45	90.42	90.44	90.56
	CuWO_4_	-	92.51	92.50	92.50	92.50	92.50	-
γ (°)	CuO	90						-
	WO_3_	-	90					
	CuWO_4_	-	82.78	82.79	82.78	82.78	82.79	-
Chi ${(\chi }^{2})$		1.28	1.78	1.85	1.48	1.92	2.81	1.70

**Table 3:** IR band assignment for the vibrational bands.

	Wavenumber Range (cm⁻¹)	Assignation	Reference
$v_{1}$	555–560	Metal–oxygen–metal bond vibrations (W-O-W, Cu-O-Cu)	[41,42]
$v_{2}$	590–605		
$v_{3}$	620–650	W-O-W stretch vibration modes	[14]
$v_{4}$	670–720	Vibration of O-W-O-W-O flexion	[43]
$v_{5}$	800–810	O-W-O stretch modes	[14]
$v_{6}$	870–920	W-O bond stretch mode in the octahedral WO_6_ structure (CuWO_4_)	[29,44,45]

**Table 4:** Particle size distributions of the samples.

Sample	Small Particle Size (nm)	Large Particle Size (nm)
CuO	130.8 ± 30.6	-
Cu80:W20	80.9 ± 28.3	-
Cu60:W40	100.5 ± 63.3	407.5 ± 95.2
Cu50:W50	96.3 ± 47.9	408.8 ± 80.6
Cu40:W60	84.9 ± 24.9	407.1 ± 81.2
Cu20:W80	82.6 ± 45.9	426.5 ± 159.3
WO_3_	99.7 ± 47.5	-

**Table 5:** Variations in the direct and indirect optical band gaps of the samples.

	Direct Gap (eV)	Indirect Gap (eV)	
	$Eg_{1}$	$Eg_{1}$	$Eg_{2}$
CuO	2.44	1.28	-
Cu80:W20	2.68	1.39	-
Cu60:W40	2.43	1.48	1.62
Cu50:W50	2.45	1.48	1.60
Cu40:W60	2.46	1.33	1.93
Cu20:W80	2.72	-	2.04
WO_3_	2.96	-	2.44

## Data Availability

The original contributions presented in this study are included in the article. Further inquiries can be directed to the corresponding author.

## References

[1] Hao R., Sun J., Liu R., Zhao H., Yao Z., Wang H., Hao Z. (2024). Emission characteristics, environmental impact, and health risk assessment of volatile organic compounds (VOCs) during manicure processes. Sci. Total Environ..

[2] Dong P., Hou G., Xi X., Shao R., Dong F. (2017). WO3-based photocatalysts: Morphology control, activity enhancement and multifunctional applications. Environ. Sci. Nano.

[3] Quan H., Gao Y., Wang W. (2020). Tungsten oxide-based visible light-driven photocatalysts: Crystal and electronic structures and strategies for photocatalytic efficiency enhancement. Inorg. Chem. Front..

[4] Adhikari S., Kim D.-H., Madras G., Sarkar D. (2018). Understanding the morphological effects of WO3 photocatalysts for the degradation of organic pollutants. Adv. Powder Technol..

[5] Gomis-Berenguer A., Celorrio V., Iniesta J., Fermin D.J., Ania C.O. (2016). Nanoporous carbon/WO3 anodes for an enhanced water photooxidation. Carbon.

[6] Wang L., Hu H., Xu J., Zhu S., Ding A., Deng C. (2019). WO3 nanocubes: Hydrothermal synthesis, growth mechanism, and photocatalytic performance. J. Mater. Res..

[7] Abe R., Takami H., Murakami N., Ohtani B. (2008). Pristine simple oxides as visible light driven photocatalysts: Highly efficient decomposition of organic compounds over platinum-loaded tungsten oxide. J. Am. Chem. Soc..

[8] Dursun S., Kaya İ.C., Kocabaş M., Akyildiz H., Kalem V. (2020). Visible light active heterostructured photocatalyst system based on CuO plate-like particles and SnO _2_ nanofibers. Int. J. Appl. Ceram. Technol..

[9] Çinar B., Keri̇moğlu I., Tönbül B., Demi̇rbüken A., Dursun S., Cihan Kaya I., Kalem V., Akyildiz H. (2020). Hydrothermal/electrospinning synthesis of CuO plate-like particles/TiO_2_ fibers heterostructures for high-efficiency photocatalytic degradation of organic dyes and phenolic pollutants. Mater. Sci. Semicond. Process..

[10] Li X., Yu J., Jaroniec M. (2016). Hierarchical photocatalysts. Chem. Soc. Rev..

[11] Alizadeh N., Salimi A., Hallaj R., Fathi F., Soleimani F. (2019). CuO/WO3 nanoparticles decorated graphene oxide nanosheets with enhanced peroxidase-like activity for electrochemical cancer cell detection and targeted therapeutics. Mater. Sci. Eng. C.

[12] Alali K.T., Liu J., Aljebawi K., Liu P., Chen R., Li R., Zhang H., Zhou L., Wang J. (2019). Electrospun n-p WO3/CuO heterostructure nanofibers as an efficient sarin nerve agent sensing material at room temperature. J. Alloys Compd..

[13] Wang C., Tang J., Zhang X., Qian L., Yang H. (2018). WO3 nanoflakes decorated with CuO clusters for enhanced photoelectrochemical water splitting. Prog. Nat. Sci. Mater. Int..

[14] Dursun S., Koyuncu S.N., Kaya İ.C., Kaya G.G., Kalem V., Akyildiz H. (2020). Production of CuO–WO3 hybrids and their dye removal capacity/performance from wastewater by adsorption/photocatalysis. J. Water Process Eng..

[15] Wang H., Xiao M., Wang Z., Chen X., Dai W., Fu X. (2023). Visible photocatalytic hydrogen production from CH3OH over CuO/WO3: The effect of electron transfer behavior of the adsorbed CH3OH. Chem. Eng. J..

[16] He M., Xie L., Zhao X., Hu X., Li S., Zhu Z.-G. (2019). Highly sensitive and selective H2S gas sensors based on flower-like WO3/CuO composites operating at low/room temperature. J. Alloys Compd..

[17] Zappa D., Galstyan V., Kaur N., Munasinghe Arachchige H.M.M., Sisman O., Comini E. (2018). “Metal oxide—Based heterostructures for gas sensors”—A review. Anal. Chim. Acta.

[18] Danish M.S.S., Estrella-Pajulas L., Alemaida I., Lisin A., Moiseev N., Ahmadi M., Nazari M., Wali M., Zaheb H., Senjyu T. (2021). Photocatalytic Applications of Metal Oxides for Sustainable Environmental Remediation. Met.—Open Access Metall. J..

[19] Yu W., Sun Y., Zhang T., Zhang K., Wang S., Chen X., Dai N. (2016). CuO/WO3 Hybrid Nanocubes for High-Responsivity and Fast-Recovery H2S Sensors Operated at Low Temperature. Part. Part. Syst. Charact..

[20] Li J., Hu S., Liu S., Hou S., Li L., Huang J. (2024). In situ fabrication of WO3/CuWO4/CuO heterojunction photoanode for boosted interfacial charge transfer and enhanced photoelectrochemical water splitting. Int. J. Hydrog. Energy.

[21] Raba-Páez A.M., Malafatti J.O.D., Parra-Vargas C.A., Paris E.C., Rincón-Joya M. (2020). Effect of tungsten doping on the structural, morphological and bactericidal properties of nanostructured CuO. PLoS ONE.

[22] Huang J., Wan Q. (2009). Gas Sensors Based on Semiconducting Metal Oxide One-Dimensional Nanostructures. Sensors.

[23] Gaona I.M.S., Mendoza M.C., Vargas C.A.P. (2023). Structural and Magnetic Properties of Nd3Ba5Cu8O18+ẟ Superconductor. J. Low Temp. Phys..

[24] Saavedra Gaona I.M., Supelano G.I., Suarez Vera S.G., Fonseca L.C.I., Castaneda Mendoza M., Sánchez Saenz C.L., Izquierdo J.L., Gómez A., Morán O., Parra Vargas C.A. (2024). Magnetic and electrical behaviour of Yb substitution on Bi1-*x*Yb*x*FeO3 (0.00 < *x* < 0.06) ceramic system. J. Magn. Magn. Mater..

[25] Pallavolu M.-R., Banerjee A.-N., Joo S.-W. (2023). Battery-Type Behavior of Al-Doped CuO Nanoflakes to Fabricate a High-Performance Hybrid Supercapacitor Device for Superior Energy Storage Applications. Coatings.

[26] Jansanthea P., Inyai N., Chomkitichai W., Ketwaraporn J., Ubolsook P., Wansao C., Wanaek A., Wannawek A., Kuimalee S., Pookmanee P. (2024). Green synthesis of CuO/Fe2O3/ZnO ternary composite photocatalyst using grape extract for enhanced photodegradation of environmental organic pollutant. Chemosphere.

[27] Abdelkarem K., Saad R., Ahmed A.M., Fathy M.I., Shaban M., Hamdy H. (2023). Efficient room temperature carbon dioxide gas sensor based on barium doped CuO thin films. J. Mater. Sci..

[28] Kang M., Liang J., Wang F., Chen X., Lu Y., Zhang J. (2020). Structural design of hexagonal/monoclinic WO3 phase junction for photocatalytic degradation. Mater. Res. Bull..

[29] Reis L.R.M., Costa M.J.S., Oliveira Y.L., Santos R.S., Sczancoski J.C., Cavalcante L.S. (2024). Structure, optical, colorimetric, and supercapacitor properties of anode α-CuWO4 crystals. Mater. Lett..

[30] Ohyama J., Iwai H., Takahashi D., Tsushida M., Machida M., Nishimura S., Takahashi K. (2024). Improved Catalytic Partial Oxidation of Methane via Lattice Oxygen Modification on Supported Copper Oxide Catalyst System. ChemCatChem.

[31] Zou Z., Zhao Z., Zhang Z., Tian W., Yang C., Jin X., Zhang K. (2023). Room-Temperature Optoelectronic Gas Sensor Based on Core–Shell g-C3N4@WO3 Heterocomposites for Efficient Ammonia Detection. Anal. Chem..

[32] Jeong Y., Hong S., Jung G., Shin W., Lee C., Park J., Kim D., Lee J.-H. (2023). Effects of oxygen gas in the sputtering process of the WO3 sensing layer on NO2 sensing characteristics of the FET-type gas sensor. Solid-State Electron..

[33] Murugesan S., Thirumurugesan R., Mohandas E., Parameswaran P. (2019). X-ray diffraction Rietveld analysis and Bond Valence analysis of nano titania containing oxygen vacancies synthesized via sol-gel route. Mater. Chem. Phys..

[34] Cuervo Farfán J.A. (2021). Producción y Propiedades Físicas de Nuevas Perovskitas Complejas del Tipo RAMOX (R = La, Nd, Sm, Eu; A = Sr, Bi; M = Ti, Mn, Fe). Ph.D. Thesis.

[35] Li X., Fu L., Karimi-Maleh H., Chen F., Zhao S. (2024). Innovations in WO3 gas sensors: Nanostructure engineering, functionalization, and future perspectives. Heliyon.

[36] Sen S.K., Dutta S., Paik L., Paul T.C., Manir M.S., Hossain M., Hossain M.N. (2021). Dy-doped MoO3 nanobelts synthesized via hydrothermal route: Influence of Dy contents on the structural, morphological and optical properties. J. Alloys Compd..

[37] Sutapa I.W., Wahid Wahab A., Taba P., Nafie N.L. (2018). Dislocation, crystallite size distribution and lattice strain of magnesium oxide nanoparticles. J. Phys. Conf. Ser..

[38] Jia Y., Zhou K., Sun W., Ding M., Wang Y., Kong X., Jia D., Wu M., Fu Y. (2024). Enhancement mechanisms of mechanical, electrical and thermal properties of carbon nanotube-copper composites: A review. J. Mater. Res. Technol..

[39] Hegde V.N., V M.V., M P.T., C H.B. (2024). Study on structural, morphological, elastic and electrical properties of ZnO nanoparticles for electronic device applications. J. Sci. Adv. Mater. Devices.

[40] Mariammal R.N., Ramachandran K., Kalaiselvan G., Arumugam S., Renganathan B., Sastikumar D. (2013). Effect of magnetism on the ethanol sensitivity of undoped and Mn-doped CuO nanoflakes. Appl. Surf. Sci..

[41] Vanasundari K., Ponnarasi P., Mahalakshmi G. (2024). A eco-friendly, green synthesis of Ag loaded WO3/rGO nanocomposites for effective UV light photocatalytic degradation of 4-nitrophenol and antimicrobial activity. Diam. Relat. Mater..

[42] Fatima R., Warsi M.F., Sarwar M.I., Shakir I., Agboola P.O., Aly Aboud M.F., Zulfiqar S. (2021). Synthesis and Characterization of Hetero-metallic Oxides-Reduced Graphene Oxide Nanocomposites for Photocatalytic Applications. Ceram. Int..

[43] Capeli R.A., Belmonte T., Caierão J., Dalmaschio C.J., Teixeira S.R., Mastelaro V.R., Chiquito A.J., Teodoro M.D., Domenegueti J.F.M., Longo E. (2021). Effect of hydrothermal temperature on the antibacterial and photocatalytic activity of WO3 decorated with silver nanoparticles. J. Sol-Gel Sci. Technol..

[44] Pourmortazavi S.M., Rahimi-Nasrabadi M., Khalilian-Shalamzari M., Ghaeni H.R., Hajimirsadeghi S.S. (2014). Facile Chemical Synthesis and Characterization of Copper Tungstate Nanoparticles. J. Inorg. Organomet. Polym..

[45] Sreekanth T.V.M., Prasad K., Yoo J., Kim J., Yoo K. (2023). CuWO4 as a cost-effective electrocatalyst for urea oxidation reaction. Inorg. Chem. Commun..

[46] Thanh N.T.K., Maclean N., Mahiddine S. (2014). Mechanisms of Nucleation and Growth of Nanoparticles in Solution. Chem. Rev..

[47] Xue S., Cao S., Huang Z., Yang D., Zhang G. (2021). Improving Gas-Sensing Performance Based on MOS Nanomaterials: A Review. Materials.

[48] Mohammed Harshulkhan S., Janaki K., Velraj G., Sakthi Ganapthy R., Nagarajan M. (2016). Effect of Ag doping on structural, optical and photocatalytic activity of tungsten oxide (WO3) nanoparticles. J. Mater. Sci: Mater. Electron..

[49] Wang Z., Wang X., Wang H., Chen X., Dai W., Fu X. (2020). The role of electron transfer behavior induced by CO chemisorption on visible-light-driven CO conversion over WO3 and CuWO4/WO3. Appl. Catal. B Environ..

[50] Landi S., Segundo I.R., Freitas E., Vasilevskiy M., Carneiro J., Tavares C.J. (2022). Use and misuse of the Kubelka-Munk function to obtain the band gap energy from diffuse reflectance measurements. Solid State Commun..

[51] Asiri A.M., Nawaz T., Tahir M.B., Fatima N., Khan S.B., Alamry K.A., Alfifi S.Y., Marwani H.M., Al-Otaibi M.M., Chakraborty S. (2021). Fabrication of WO3 based nanocomposites for the excellent photocatalytic energy production under visible light irradiation. Int. J. Hydrog. Energy.

[52] Escobedo-Morales A., Ruiz-López I.I., Ruiz-Peralta M.D., Tepech-Carrillo L., Sánchez-Cantú M., Moreno-Orea J.E. (2019). Automated method for the determination of the band gap energy of pure and mixed powder samples using diffuse reflectance spectroscopy. Heliyon.

[53] Jubu P.R., Obaseki O.S., Yam F.K., Stephen S.M., Avaa A.A., McAsule A.A., Yusof Y., Otor D.A. (2023). Influence of the secondary absorption and the vertical axis scale of the Tauc’s plot on optical bandgap energy. J. Opt..

[54] Jubu P.R., Obaseki O.S., Ajayi D.I., Danladi E., Chahrour K.M., Muhammad A., Landi S., Igbawua T., Chahul H.F., Yam F.K. (2024). Considerations about the determination of optical bandgap from diffuse reflectance spectroscopy using the tauc plot. J. Opt..

[55] Zhang H., Yilmaz P., Ansari J.O., Khan F.F., Binions R., Krause S., Dunn S. (2015). Incorporation of Ag nanowires in CuWO4 for improved visible light-induced photoanode performance. J. Mater. Chem. A.

[56] Raba-Páez A.M., Malafatti J.O.D., Parra-Vargas C.A., Paris E.C., Rincón-Joya M. (2021). Structural evolution, optical properties, and photocatalytic performance of copper and tungsten heterostructure materials. Mater. Today Commun..

[57] Morales-Morales G., Manzanares-Martinez J. (2022). Enlargement of band gaps on thermal wave crystals by using heterostructures. Results Phys..

[58] Yao Y., Sang D., Zou L., Wang Q., Liu C. (2021). A Review on the Properties and Applications of WO3 Nanostructure-Based Optical and Electronic Devices. Nanomaterials.

[59] Wei Z., Zhou Q., Lu Z., Xu L., Gui Y., Tang C. (2019). Morphology controllable synthesis of hierarchical WO3 nanostructures and C2H2 sensing properties. Phys. E Low-Dimens. Syst. Nanostructures.

[60] Wang L., Cheng S., Wu C., Pei K., Song Y., Li H., Wang Q., Sang D. (2017). Fabrication and high temperature electronic behaviors of n-WO3 nanorods/p-diamond heterojunction. Appl. Phys. Lett..

[61] Shen Z., Peng Z., Zhao Z., Fu X. (2018). Nonlinear current-voltage characteristics of WO3-x nano-/micro-rods. Solid State Sci..

[62] Krishna K.G., Parne S., Pothukanuri N., Kathirvelu V., Gandi S., Joshi D. (2022). Nanostructured metal oxide semiconductor-based gas sensors: A comprehensive review. Sens. Actuators A Phys..

[63] Cho S.-Y., Jang D., Kang H., Koh H.-J., Choi J., Jung H.-T. (2019). Ten Nanometer Scale WO3/CuO Heterojunction Nanochannel for an Ultrasensitive Chemical Sensor. Anal. Chem..

[64] Goel N., Kunal K., Kushwaha A., Kumar M. (2023). Metal oxide semiconductors for gas sensing. Eng. Rep..

[65] Karnati P., Akbar S., Morris P.A. (2019). Conduction mechanisms in one dimensional core-shell nanostructures for gas sensing: A review. Sens. Actuators B Chem..

[66] Gou X., Wang G., Yang J., Park J., Wexler D. (2008). Chemical synthesis, characterisation and gas sensing performance of copper oxide nanoribbons. J. Mater. Chem..

[67] Raksa P., Gardchareon A., Chairuangsri T., Mangkorntong P., Mangkorntong N., Choopun S. (2009). Ethanol sensing properties of CuO nanowires prepared by an oxidation reaction. Ceram. Int..

[68] Ahmad M.Z., Kang J.H., Sadek A.Z., Moafi A., Sberveglieri G., Wlodarski W. (2012). Synthesis of WO3 Nanorod based Thin Films for Ethanol and H2 Sensing. Procedia Eng..

[69] Yang F., Wang F., Guo Z. (2018). Characteristics of binary WO3@CuO and ternary WO3@PDA@CuO based on impressive sensing acetone odor. J. Colloid Interface Sci..

